# A new species of *Miconia* (Melastomataceae, Miconieae) from the Ecuador-Peru border

**DOI:** 10.3897/phytokeys.12.3027

**Published:** 2012-04-25

**Authors:** Carmen Ulloa Ulloa, David A. Neill, Olivia A. Dudek

**Affiliations:** 1 Missouri Botanical Garden, P.O. Box 299, St. Louis, MO 63166-0299, USA; 2 Universidad Estatal Amazónica, Puyo, Pastaza, Ecuador; 3 Washington University, 6985 Snow Way Drive, St. Louis, MO 63130-4400, USA

**Keywords:** Cordillera del Cóndor, Ecuador, endemism, IUCN, Melastomataceae, *Miconia*, Peru

## Abstract

*Miconia machinazana* C.Ulloa & D.A. Neill, **sp. nov.**,a new species of Melastomataceae from the Ecuador-Peru border is described and illustrated. It is characterized by the narrow, decussate leaves, dense reddish brown indument, small flowers in short panicles, pale yellow petals, and anthers opening by two large terminal pores.

## Abstract (es)

Se describe e ilustra *Miconia machinazana* C. Ulloa & D.A. Neill, **sp. nov.**, una nueva especie de Melastomataceae de la frontera ecuatoriano-peruana, caracterizada por las hojas angostas, decusadas, el denso indumento café-rojizo, las flores pequeñas en panículas cortas, los pétalos de un amarillo pálido y las anteras abriéndose por dos poros anchos terminales.

## Introduction

*Miconia* Ruiz & Pav., is a megadiverse genus, the largest in the family Melastomataceae, and comprises some 1050 Neotropical species (Goldenberg et al. 2008). Some 250 species have been recorded for Ecuador (Wurdack 1980, Renner 1999, Cotton 2000, Ulloa Ulloa and Homeier 2008), occurring from sea level to ca. 4000 m. Within the Melastomataceae, *Miconia* belongs to the Neotropical tribe Miconieae DC. *Miconia* is paraphyletic, and its current circumscription is rather arbitrary. *Miconia* is distinguished only by plesiomorphic characters found elsewhere in the tribe, and by eliminating other genera (Goldenberg et al. 2008, Michelangeli et al. 2004). As currently understood, the genus is characterized by its woody habit, terminal inflorescences, hypanthium not apically constricted, calyx of small lobes not forming a circumscissile cap, anthers without a bifurcation at the base, and a fleshy fruit (Cogniaux 1891, Judd and Skean 1991, Goldenberg et al. 2008).

Explorations in the remote Cordillera del Cóndor in southern Ecuador yielded a shrub erroneously identified in the field as the genus *Myrteola* O. Berg (Myrtaceae) due to the decussate, coriaceous, small, linear leaves reminiscent of *Myrteola phylicoides* (Benth.) Landrum. The leaf arrangement and the non-apparent classic checkerboard venation, of many species in the Melastomataceae led to that mistake. However, the habit of this plant is characteristic of a few high Andean species of *Miconia*, including the widespread *Miconia salicifolia* (Naudin) Naudin,to which this plant shows a similar habit. Further study has revealed unique features that lead us to propose it as a new species of *Miconia*.

## Methods

Herbarium and laboratory work involved taking measurements of the vegetative parts from the dry herbarium specimens; the flowers were rehydrated before taking measurements under a dissecting scope. Seeds were sputter coated with gold/palladium and photographed with a scanning electron microscope (JEOL JCM-5000). Herbarium specimens were consulted and compared at HA, K, LOJA, MA, MO, QCA, and QCNE; necessary herbarium specimens were requested on loan, and additional material was consulted over the internet in various virtual herbaria (COL, NY, US, JStor Plant Science types).

## Taxonomic treatment

### Miconia machinazana

C.Ulloa & D.A.Neill sp. nov.

urn:lsid:ipni.org:names:77118901-1

http://species-id.net/wiki/Miconia_machinazana

**Figure 1.**
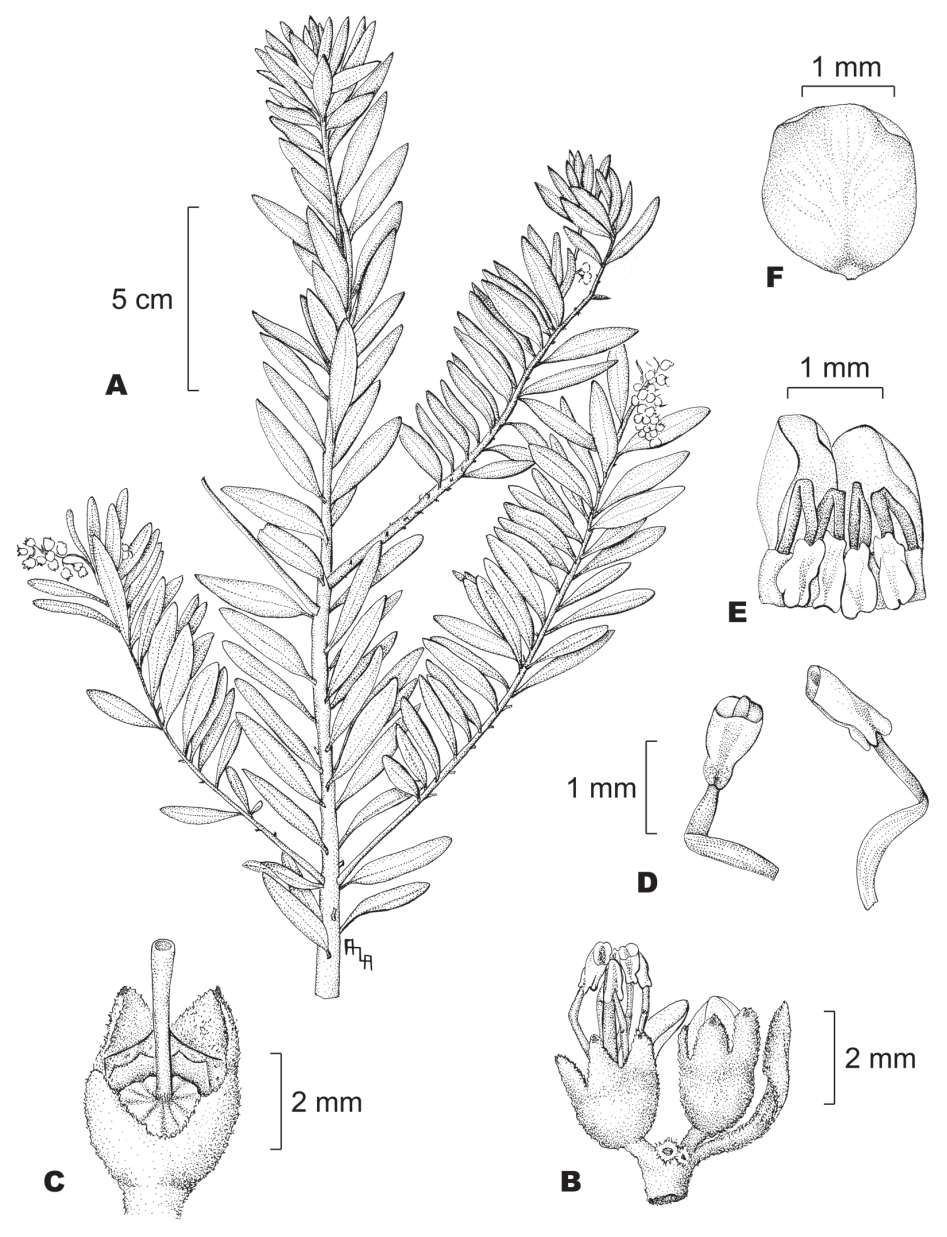
*Miconia machinazana* C. Ulloa & D.A. Neill, **A** fertile branch **B** partial inflorescence and flower bract **C** young fruit crowned by calyx lobes and style **D** stamens, ventral (left) and lateral views **E** young stamens folded inside a partial flower bud **F** petal, ventral view. Line drawing by A.L.Arbeláez; voucher Neill & Kajekai 16909.

Figs 1 [Fig F2] [Fig F3] –4

#### Note.

Haec species ad *Miconiam* sect. *Cremanium* (D.Don) Hook.f. pertinens, a congeneris sect. *Chaenopleurae* (DC.) Hook.f. floribus pentameris differt; intra sect. *Cremanium* a *Miconia rigente* Naudin foliis minoribus (usque ad 35.1 mm vs. 60 mm longis) petalis luteolis (vs. albis et roseis) atque foliis 1- (vs. 3-)nerviis, a *Miconia ledifolia* (DC.) Naudin foliis latioribus (usque ad 10.5 mm vs. 4 mm latis) atque fructu majore (2.4–3.9 × 2.8–3.8 mm in *Miconia ledifolia*) seminibus pluribus (usque ad 9 in *Miconia ledifolia*) differt.

#### Type.

**Ecuador**. Zamora-Chinchipe: Paquisha, Cordillera del Cóndor. The Machinaza plateau. About 500 m W of the Ecuador-Peru international border, near end of trail from Paquisha Alto military post. 03°54'06"S, 78°28'57"W, 2315 m, 23 June 2009 (fl, fr), D.A.Neill & C.Kajekai 16909 (holotype MO!; isotypes AAU!, CAS!, LOJA!, M!, NY!, QCNE!).

#### Description.

Small, profusely branched shrub 0.5–1.2 m tall; internodes 1.5–7.0 × 1.2–2.4 mm. A thick indument of pinoid trichomes densely covering and totally concealing the surface of branches, petioles, both surfaces of young leaves, bracts, pedicels, hypanthium, calyx lobes outside surface, and fruits, the indument reddish-brown (cinnamon) colored on young parts and becoming darker, maroon-brown, and caducous on older organs, leaving the base of older branches and the adaxial surface of leaves with scattered trichomes. Leaves decussate, the petiole erect and nearly parallel to the stem, and the blade ascending at an angle of 90-120º with the petiole, 11.8–35.1 × 2.2–10.5 mm, narrowly elliptic to narrowly lanceolate, coriaceous, with 15–22 pairs of faintly visible nerves adaxially, the base acute, the margins revolute and remotely crenate, the minute teeth dark, the adaxial surface dark green with scattered pinoid whitish trichomes on the surface and covering the midrib, the abaxial surface concealed by the indument, the apex bluntly acute and mucronate; petiole 1.6–4.5 mm. Inflorescences 10–20 mm, panicles, terminal at the tip of the branches or on short lateral branches, 1–3 flowers open at a time; bracts 3–9 mm, spatulate, persistent. Hypanthium 1.0–2.4 mm, campanulate, maroon red, glabrous inside. Flowers 5-merous; calyx lobes ca. 1.2 × 1.2 mm, maroon red and glabrous adaxially, the external teeth thick, ca. 0.35 mm, projecting, concealed by the indument. Corolla pale yellow, the petals 1.3–2 × 1.5–1.7 mm, concave, the apex oblique, the margin minutely erose, the outer surface granulose. Stamens 10, slightly dimorphic in size, the filaments 2.0–2.3 mm, geniculate above the middle, twice as wide below the folding point towards the base, cream colored, tinged with pink in older flowers, the anthers 0.9–1.2 mm, 2-celled, obovate, retuse at apex, initially uniformly cream colored and later tinged with pink, opening by two broad, apical, ventrally inclined pores, the connective at the base ventrally with a blunt, bilobed appendage and dorsally with a blunt, minutely notched tooth, slightly longer than the ventral lobes; ovary 3-celled, 3/4 inferior, ridged, with a ring of pinoid trichomes at the apex, the style ca. 3.5 mm, straight, pale yellow, glabrous, the stigma clavate but not conspicuously so, pale yellow. Infructescences with up to 22 mature fruits. Berries 5–7 × 5.5–7.5 mm, nearly globose, fleshy, the surface concealed by the indument, maroon red apically, ridged and with a few trichomes at the base of the attachment of the style; seeds 15–25, globose, ca. 0.99 × 0.93 mm.

**Figure 2. F2:**
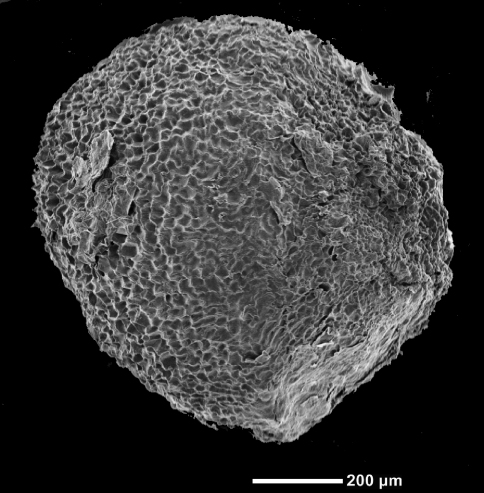
Scanning electron micrograph of a seed of *Miconia machinazana* (photograph O.A. Dudek).

**Figure 3. F3:**
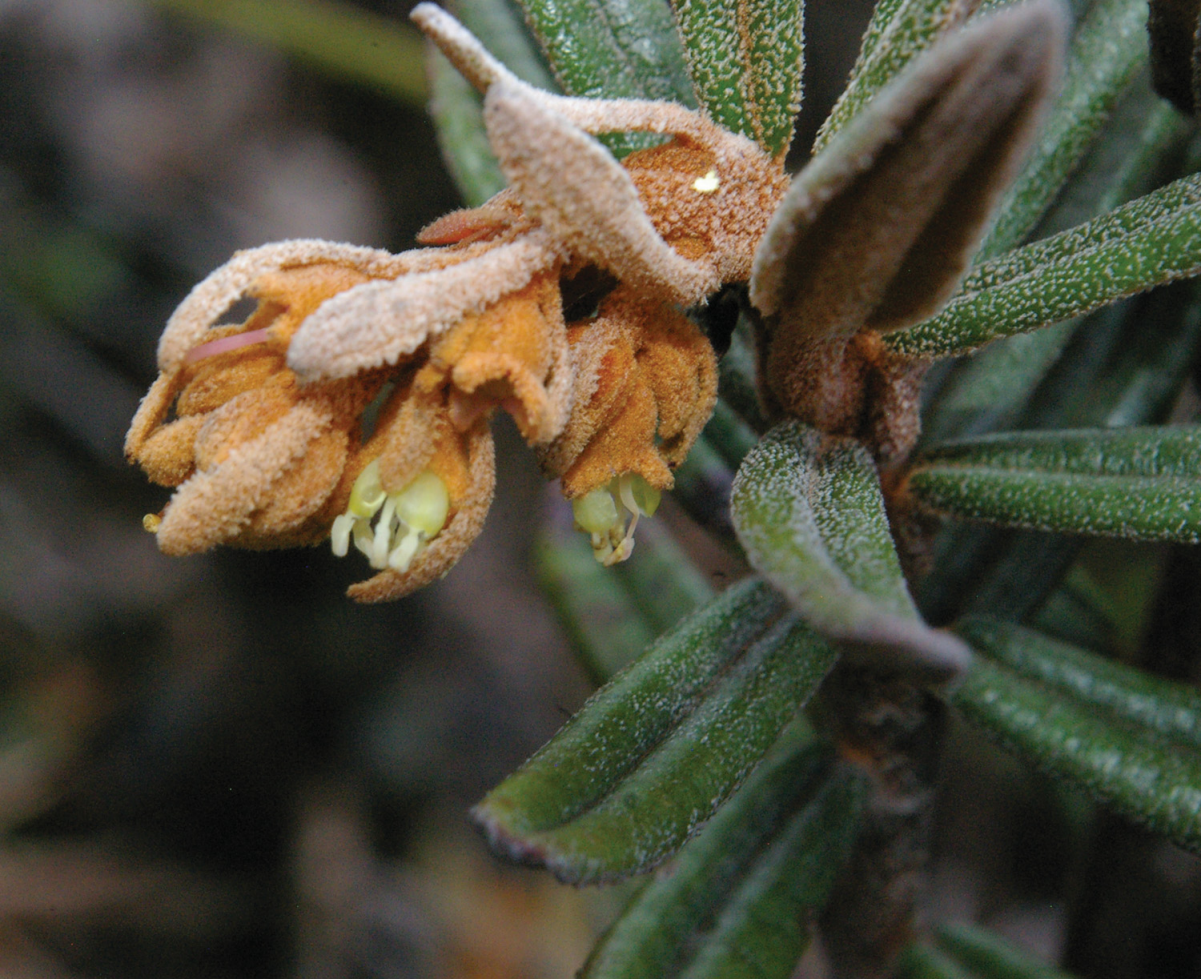
*Miconia machinazana*, flowering branch, *Neill 16909* (photograph D.A. Neill).

**Figure 4.**
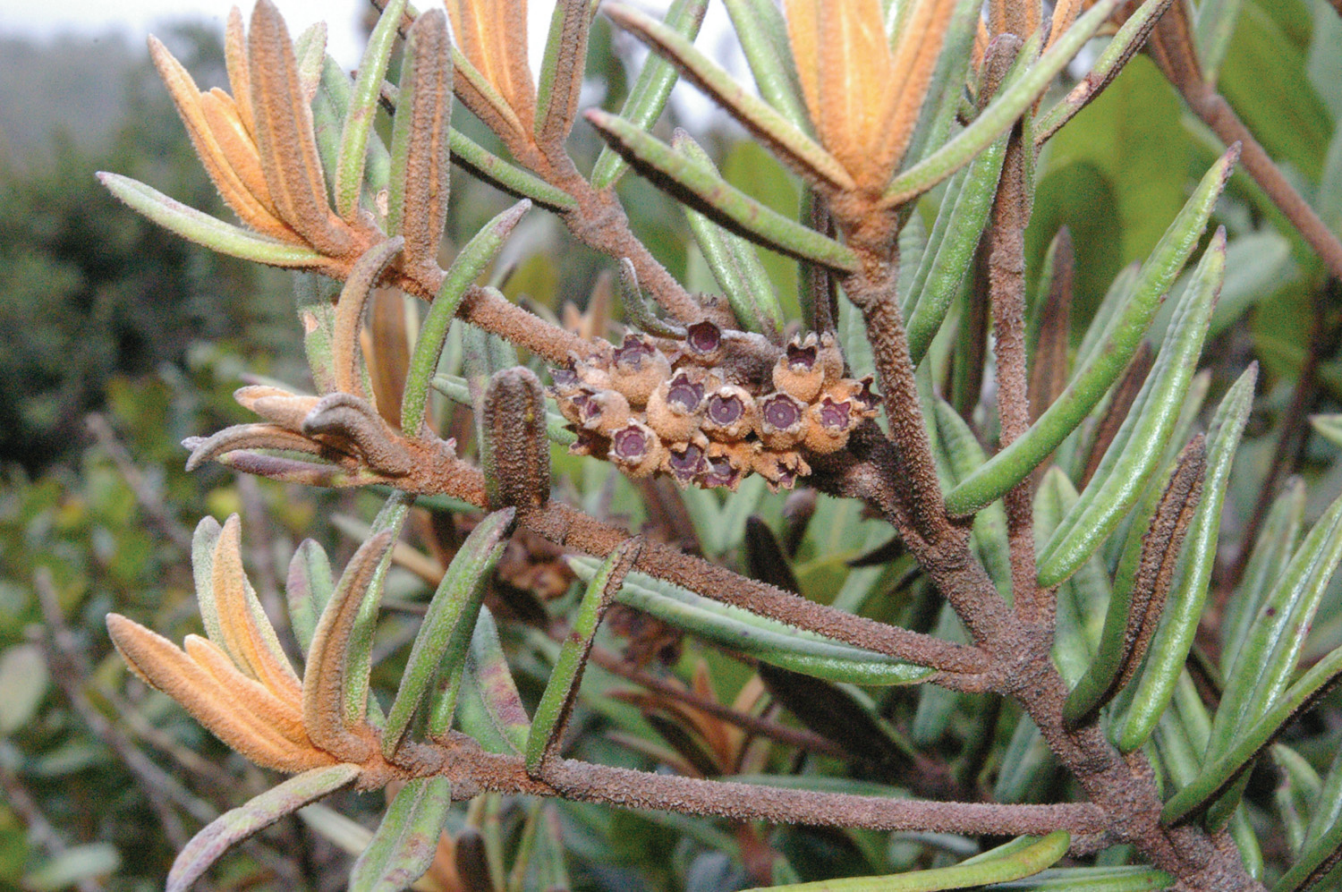
*Miconia machinazana*, fruiting branch, *Neill 16167* (photograph D.A. Neill).

#### Distribution.

**Figure 5.**
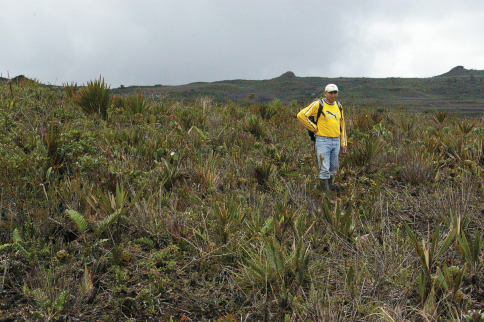
Vegetation at the summit of the Machinaza plateau, Cordillera del Cóndor, Ecuador, 2450 m (photograph D.A. Neill).

*Miconia machinazana* has only been found on the Machinaza plateau, one of the highest-elevation Hollín Sandstone plateaus in the Cóndor region between 2315 and 2420 m (Fig. 5). The area is precisely on the Ecuador-Peru international border, near the end of the trail from the Paquisha Alto military post. Since the population actually straddles the border it is recorded as occurring in the province of Zamora-Chinchipe in Ecuador and in the department of Amazonas, Peru.

#### Ecology.

The specimens collected in June have just a few open flowers and several fruits, while the specimens collected in March have abundant fruits. The Cóndor is an eastern outlier of the main Andes chain and has revealed a fascinating and unexpected biogeographical connection between the sub-Andean cordilleras and the Guayana Shield in northeastern South America (Ulloa Ulloa and Neill 2006). The soils are very nutrient-poor, and consist of a bare sandstone substrate or coarse- to medium-grained quartzite sand derived there from. The vegetation is mostly dwarf scrub, dominated by shrubs to about 0.5 m tall, with occasional small trees to four meters tall (Fig. 5). The vegetation seems to be recovering slowly from an extensive fire that occurred between 1990 and 1995, with charred woody stems in abundance on the ground.

#### Etymology.

The species name *machinazana* commemorates the name of the Machinaza plateau and river in the Cordillera del Cóndor area where this species was collected.

#### Conservation status.

*Miconia machinazana* has a restricted distribution, only known from scattered populations within a single mountain range. The Area of Occupancy (AOO) of the species is 3 km^2^ and it falls completely outside any protected area under Ecuador’s System of Protected Areas. In terms of our current knowledge, the species is assigned a provisional IUCN (2001) conservation status of Critically Endangered (IUCN SPWG 2010).

#### Paratypes.

Ecuador. Zamora-Chinchipe:Centinela del Cóndor, Cordillera del Cóndor, Machinaza plateau summit area, adjacent to obelisk-shaped border marker, at end of trail from upper Paquisha military post, precisely at Ecuador-Peru border. 03°53'50"S, 78°28'49"W, 2420 m, 15 Mar 2008 (fr), D.A.Neill & W.Quizhpe 16167 (COL, HA, K, LOJA, MO, QCA, QCNE, USM).

## Discussion

*Miconia machinazana* differs from other species of *Miconia* by the combination of the strictly decussate arrangement of very narrow, coriaceous leaves, thick reddish brown (cinnamon) indument of pinoid trichomes, pale yellow petals, the anthers opening by two large pores, and the berries with large seeds. Following Wurdack’s (1980) Flora of Ecuador key to Artificial Species Groups, this species will key out within group D, the group of species with the lower leaf surface completely concealed by the dense pubescence, and next to *Miconia ledifolia* (DC.) Naudin. In Cogniaux’s (1891) classification and following Goldenberg et al. (2008) this species belongs in *Miconia* sect. *Cremanium* (D.Don) Hook.f., that is characterized by the very short anthers with two or four, wide apical pores.

*Miconia machinazana* has pale yellow petals, yellow being a color uncommon in the tribe Miconieae (Almeda 2000, Morales-Puentes et al. 2008). This new speciesresembles in its habit the widespread *Miconia salicifolia*, however, a closer examination immediately differentiates the species belonging not only to different sections (the latter belongs to sect. *Chaenopleura* (DC.) Hook.f.), but showing differences in flower merosity, stigma shape, fruit size, and number of seeds per fruit (see Table 1). *Miconia rigens* Naudin, *Miconia ledifolia*, *Miconia stenophylla* Wurdack, and *Miconia tephrodes* Wurdack are all high-Andean shrubs with short inflorescences, crowded leaves with the lower surface totally concealed by a dense indument, and the stamens opening by two apical pores, as in *Miconia machinazana*, but the combination of characters separates each of these taxa and with the new species (see Table 1). The species here compared from sect. *Chaenopleura* have 4-merous flowers, but the flowers are 5-merous in the ones from sect. *Cremanium*. *Miconia rigens*, from Colombia’s Cordillera Oriental, is distinct by its much wider 3-nerved leaves (up to 3 cm) and longer internodes.

**Figure 6.**
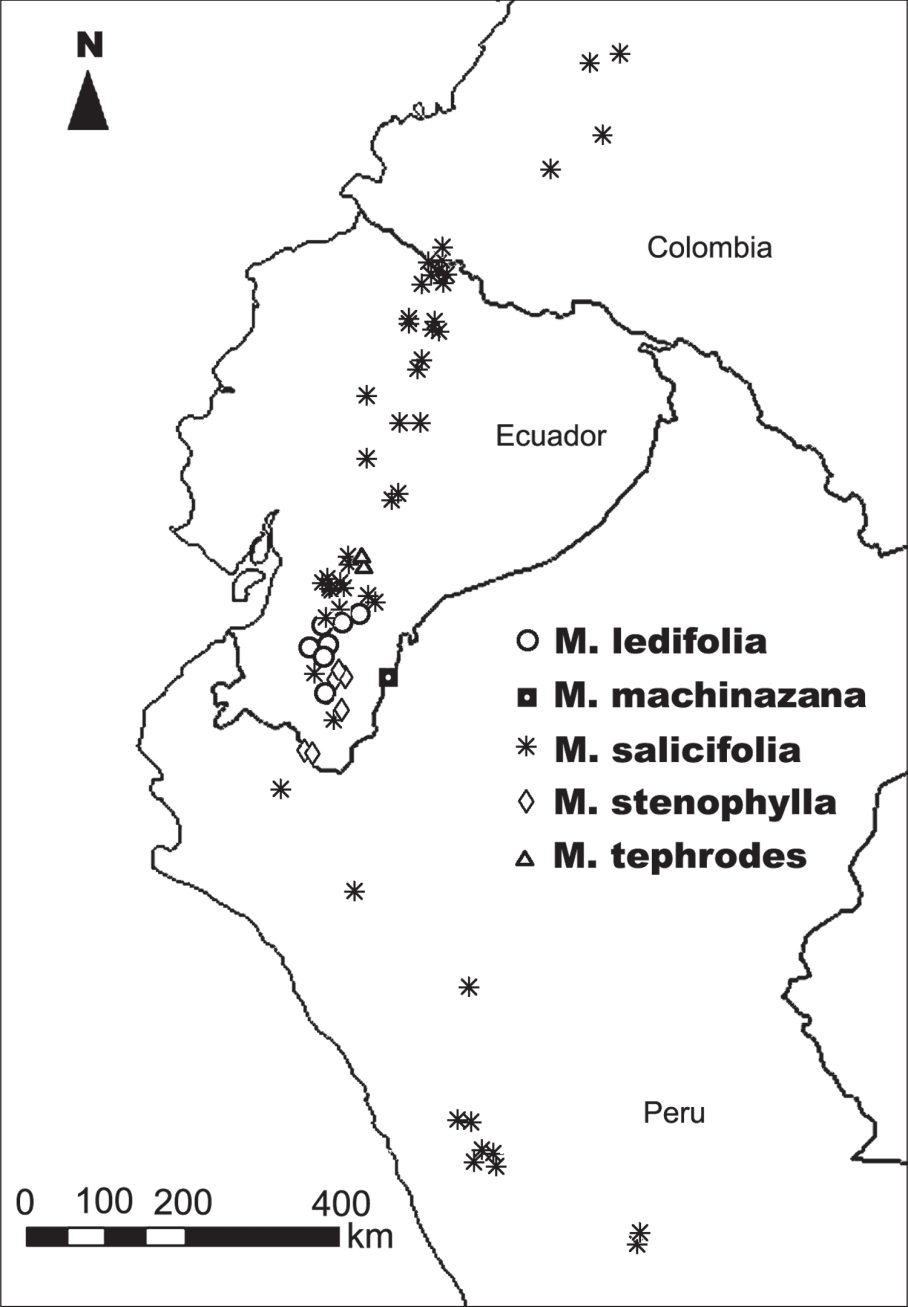
Distribution map of *Miconia ledifolia*, *Miconia machinazana*, *Miconia salicifolia* (partial), *Miconia stenophylla*, and *Miconia tephrodes* (See Table 1; for complete species distribution see www.tropicos.org).

The species compared in Table 1, apart from for *Miconia salicifolia*, are all narrow endemics. *Miconia rigens* is a seemingly rare species, restricted to the páramo of a small area of Colombia’s Boyacá Department (Fernández Alonso pers. comm.), and the rest occur in southern Ecuador, where *Miconia tephrodes* is only known from four collections from the Eastern Andean Cordillera, and *Miconia machinazana* farther east from the remote Cóndor sandstone plateau (Fig. 6).

**Table 1.** Comparison of *Miconia machinazana* and other Andean species of *Miconia* with leaf lower surface completely concealed by indument.

Characters	*Miconia machinazana*	*Micona ledifolia*	*Miconia rigens*	*Miconia salicifolia*	*Miconia stenophylla*	*Miconia tephrodes*
Internode length (mm)	1.5–7	1.8–6.4	8–14	2–7.1	2–4.3	2.6–3.4
Leaf blade length (mm)	11.8–35.1	10–15(–22)	40–60	20–50	8–14	8–15
Leaf blade width (mm)	(2.2)3–10.5	1.5–2.5(–4)	20–30	3–7	1–2	4–7
Leaf blade main nerves	1-nerved	1-nerved	3-nerved	3-nerved	1-nerved	1-nerved
Petiole length (mm)	1.6–4.5	1–3	2–4	2–5	1–2	1.5–3
Flower merosity	five	five	five	four	four	four
calyx lobes length (mm)	ca. 1.2	4–5	ca. 3	4–5	ca. 2	3–4
Calyx external teeth	thick	thick	inconspicous	not projecting	appressed	inconspicous
Petals color	pale yellow	pale yellow	white and pink	cream-white	pink	unknown
Petals length (mm)	1.3–2	1.5–1.7	ca. 2	1.7–1.8	ca. 1.4	ca. 1.1
Filaments indument	glabrous	glabrous	glabrous	moderately puberulous abaxially	apically sparsely glandular-setulose	apically sparsely glandular
Stigma shape	clavate not expanded	barely clavate-expanded	subpeltate	elongate capitate	capitate	capitate
Ovary cells and position	3-celled and 3/4 inferior	3-celled and 1/3 inferior	3-celled and 3/4 inferior	3-4 celled and 2/3 inferior	3-celled and 1/2 inferior	3-celled and 1/2 inferior
Ovary apical cone indument	ring of pinoid hairs	glabrous or sparsely furfuraceous	glabrous	stylar collar	densely glandular-puberulous	stellulate furfuraceous and glandular
Fruit length × width (mm)	5–7 × 5.5–7.5	2.4–3.9 × 2.8–3.8	4–6 × 4–6	2.5–4 × 2.5–5.4	2–3 × 2.8–3.5	1.4–2 × 1.4–2.1
Seed number per fruit	15–25	6–9	15–23	55–95	ca. 50	9–15
Country (Division)	Ecuador (Zamora-Chinchipe); Peru (Amazonas, not collected)	Ecuador (Azuay, Loja)	Colombia (Boyacá)	N Colombia to C Peru (divisions below†)	Ecuador (Azuay, Loja)	Ecuador (Azuay, Cañar, Morona-Santiago)
Elevation (m)	2315–2420	2200–3800	3105–3450	2600–4400	2500–3500	3200–3500
Section within Miconia	Cremanium	Cremanium	Cremanium	Chaenopleura	Chaenopleura	Chaenopleura
Representative specimen	Neill 16167, 16909 (MO)	Ulloa 1508 (HA, MO)	Cuatrecasas 9787 (COL, US)	Ulloa 2117 (HA, MO, QCA)	Prieto P-312 (MO)	Camp E-4872 (US)

†Colombia: Antioquia, Boyacá, Caldas, Cauca, Cundinamarca, Santander, Tolima. Ecuador: Azuay, Cañar, Carchi, Chimborazo, Cotopaxi, Imbabura, Loja, Morona-Santiago, Napo, Pichincha, Tungurahua. Peru: Ancash, Cusco, La Libertad, Lambayeque, Pasco.

## Supplementary Material

XML Treatment for Miconia machinazana
